# 30-day mortality in patients treated for brain metastases: extracranial causes dominate

**DOI:** 10.1186/s13014-022-02062-x

**Published:** 2022-05-12

**Authors:** Carsten Nieder, Luka Stanisavljevic, Siv Gyda Aanes, Bård Mannsåker, Ellinor Christin Haukland

**Affiliations:** 1 Department of Oncology and Palliative Medicine, Nordland Hospital, 8092 Bodø, Norway; 2 Department of Clinical Medicine, Faculty of Health Sciences, UiT—The Arctic University of Norway, Tromsö, Norway; 3 Department of Quality and Health Technology, Faculty of Health Sciences, SHARE—Center for Resilience in Healthcare, University of Stavanger, Stavanger, Norway

**Keywords:** Palliative radiation therapy, Stereotactic radiotherapy, Brain metastases, Prognostic factors, Biomarkers

## Abstract

**Background:**

Established prognostic models, such as the diagnosis-specific graded prognostic assessment, were not designed to specifically address very short survival. Therefore, a brain metastases-specific 30-day mortality model may be relevant. We hypothesized that in-depth evaluation of a carefully defined cohort with short survival, arbitrarily defined as a maximum of 3 months, may provide signals and insights, which facilitate the development of a 30-day mortality model.

**Methods:**

Retrospective analysis (2011–2021) of patients treated for brain metastases with different approaches. Risk factors for 30-day mortality from radiosurgery or other primary treatment were evaluated.

**Results:**

The cause of death was unrelated to brain metastases in 61%. Treatment-related death (grade 5 toxicity) did not occur. Completely unexpected death was not observed, e.g. accident, suicide or sudden cardiac death. Logistic regression analysis showed 9 factors associated with 30-day mortality (each assigned 3–6 points) and a point sum was calculated for each patient. The point sum ranged from 0 (no risk factors for death within 30 days present) to 30. The results can be grouped into 3 or 4 risk categories. Eighty-three percent of patients in the highest risk group (> 16 points) died within 30 days, and none survived for more than 2 months. However, many cases of 30-day mortality (more than half) occurred in intermediate risk categories.

**Conclusion:**

Extracranial tumor progression was the prevailing cause of 30-day mortality and few, if any deaths could be considered relatively unexpected when looking at the complete oncological picture. We were able to develop a multifactorial prediction model. However, the model’s performance was not fully satisfactory and it is not routinely applicable at this point in time, because external validation is needed to confirm our hypothesis-generating findings.

## Background

Most patients with brain metastases from extracranial primary tumors such as lung or breast cancer receive palliative treatment approaches, because the common pattern of polymetastatic spread may cause compromised performance status (PS) and eventually also limited survival, often in the range of 3–9 months [1]. Both, longer survival (typically if oligometastases are present [2]) and shorter survival may be observed, and considerable efforts have been undertaken to predict survival (nomograms, scores, online calculators [3–5]). Given that very short survival often is synonymous to active treatment in the last 30 days of life, oncologists can opt for palliative and supportive care rather than brain-directed therapy [6]. Supposing they choose brain-directed therapy, the challenge is to navigate a complex scenario of low-value care, potential overtreatment and futile, but costly procedures [7, 8].

Prognostic models familiar to many providers, such as the diagnosis-specific graded prognostic assessment (DS-GPA) [3], were not designed to specifically address very short survival. Different definitions of very short survival may be applied, including 30-day mortality, which has been evaluated in numerous oncology settings [9, 10]. General survival prediction models such as TEACHH may also be utilized [11, 12], but it is still unclear whether a brain metastases-specific 30-day mortality model should be preferred. Our group has previously studied different models that predict very short survival (not specifically focused on 30 days), but none of these was considered truly satisfactory [13–15]. The fact that many patients with poor prognosis were not identified by any model was considered a major challenge. Ideally, a model would identify all or almost all patients with very short survival, and simultaneously, patients predicted to die early would not survive long enough to benefit from active treatment. In other words, both over- and undertreatment should be avoided, because shortening survival by withholding treatment would be a serious threat too.

These reflections are also applicable to the recently introduced LabPS score (blood test results and PS) [14], where the group with the poorest prognosis (3 or 3.5 points; maximum survival 2.1 months) was very small (4% of all patients in the study). Most patients with comparably short survival had a lower point sum. The LabPS score failed to outperform the previously proposed extracranial-graded prognostic assessment score (EC-GPA) [15]. Median survival was 0.7 months in the worst prognostic group of the latter score, with a hazard ratio for death of 44 (95% confidence interval (CI), 6–340) compared to the best group. However, many patients with short survival were not assigned to the worst group. After these previous, only partially successful studies that included all-comers, we changed our methodology, went back to the drawing board and hypothesized that in-depth evaluation of a carefully defined cohort with short survival, arbitrarily defined as a maximum of 3 months, may provide signals and insights, which facilitate the development of viable 30-day mortality models.

## Patients and methods

A previously described, continuously updated quality-of-care database covering all adult patients with brain metastases at the authors’ institution, which employs electronic health records containing detailed follow-up information, was utilized [14]. The cohort was limited to patients who survived ≤ 3.0 months from commencing their first treatment (start of primary whole-brain radiotherapy (WBRT), date of radiosurgery (SRS), start of systemic treatment etc.), whether treatment was completed or not (intention-to-treat). The study did not include patients who received their second treatment, e.g. delayed salvage WBRT after previous SRS. Patients with leptomeningeal central nervous system metastases and those managed with best supportive care after diagnosis of brain metastases were not included. The database includes patients with solid tumors only, whereas those with leukemia and lymphoma are excluded. For the purpose of this exploratory, hypothesis-generating study, a cohort size of *n* = 100 was deemed appropriate. We felt that there was no solid fundament for statistical hypotheses or power calculations in the planning process. Starting with recent patients treated in 2021, backward inclusion of consecutive patients was employed. The target size of 100 patients was reached when including patients treated in the year 2011.

Besides established baseline parameters such as age, sex, number of brain metastases and Karnofsky PS, blood test results were included (hemoglobin, platelets, C-reactive protein, albumin, lactate dehydrogenase (LDH); the components of the LabBM score, which was assigned as originally recommended [16]). These were also employed to assign the LDH/albumin-based extracranial score (EC-S) [15]. The pattern and number of extracranial sites was registered (uncontrolled primary tumor, liver, lung, bone and other extracranial metastases; standard staging considered appropriate by the treating physicians at the time of treatment, thus subject to temporal and cancer-type-related variation [17]). Most likely, the blood test results mirror the overall burden of disease, including lesions not correctly identified on radiological examinations [16, 17]. The total number of brain metastases was derived from magnetic resonance imaging (MRI) reports. Cumulative lesion volume was not available.

Cause of death was recorded in order to account for surprising, unpredictable events such as accidents. Chi-square tests were employed to identify factors predicting 30-day mortality (30 days from SRS, first fraction of WBRT, day 1 of chemotherapy etc.). The latter were further examined in multinominal logistic regression analysis. Statistical significance was defined as *p* < 0.05 in two-sided tests. The methods employed by Rades et al. were utilized to calculate a point sum reflective of 30-day mortality [18, 19]. For example, a risk factor associated with 50% 30-day mortality was assigned 5 points, while 3 points were assigned for a factor associated with 30% 30-day mortality. The predictive accuracy of our model was evaluated using Harrell’s concordance index (Harell’s C). Harrell’s C shows perfect concordance if the value is 1, whilst a value of 0.5 indicates completely random concordance (an unserviceable model in other words).

## Results

The most common treatment approach was WBRT (30 Gy in 10 fractions, 64%; 20 Gy in 5 fractions, 12%). Eighteen percent of all patients failed to complete their prescribed treatment. Common tumor types included non-small cell lung cancer (NSCLC, 42%), malignant melanoma (12%) and breast cancer (11%). Detailed baseline characteristics are shown in Table 1. The 30-day mortality was 28% and an additional 39% died between 31 and 60 days.

**Table 1:** Patient characteristics, *n* = 100

Baseline parameter	Number (= %)
Sex	
Female sex	46
Male sex	54
Tumor type	
Non-small cell lung cancer	42
Breast cancer, triple negative	3
Breast cancer, Her2 positive	4
Breast cancer, other	4
Malignant melanoma	12
Small cell lung cancer	9
Renal cell cancer	8
Colorectal cancer	10
Other gastrointestinal cancer	5
Other primary tumors (bladder, head/neck)	3
Extracranial disease	
No extracranial metastases	9
Extracranial metastases	91
Bone metastases	37
Liver metastases	38
Lung/pleura metastases	56
Controlled primary tumor	55
Uncontrolled primary tumor*	45
Active organ sites incl. uncontrolled primary tumor: 0	5
Active sites: 1**	16
Active sites: 2	25
Active sites: 3	31
Active sites: 4	17
Active sites: > 4	6
Brain metastases	
Single brain metastasis	12
Two or three brain metastases	21
Four or five brain metastases	19
Six to ten brain metastases	27
More than ten brain metastases	21
Synchronous brain metastases	24
Metachronous brain metastases, within 12 months	37
Metachronous brain metastases, 13–24 months	11
Metachronous brain metastases, 25–36 months	11
Metachronous brain metastases, 37–60 months	8
Metachronous brain metastases, > 60 months	9
Asymptomatic brain metastases	9
Symptom response to steroids	64
No response to steroids	27
Largest lesion diameter ≤ 2 cm	48
Largest lesion diameter 2.1–3.0 cm	23
Largest lesion diameter 3.1–4.0 cm	19
Largest lesion diameter > 4.0 cm	10
Karnofsky performance status (KPS)	
KPS 50	14
KPS 60	30
KPS 70	44
KPS 80	8
KPS 90	4
Treatment	
Primary systemic treatment	7
Surgery with post-operative cavity radiotherapy	2
Stereotactic single fraction radiosurgery	6
Stereotactic fractionated radiotherapy	6
Whole-brain radiotherapy, 20 Gy in 5 fractions	12
Whole-brain radiotherapy, 30 Gy in 10 fracions	64
Whole-brain radiotherapy, higher dose than 30 Gy	3
Any systemic therapy after diagnosis of brain metastases	34
Age, years	
< 60	18
60–69	40
70–79	35
80–89	5
≥ 90	2
Extracranial score (EC-S; LDH, albumin, extracranial involvement of at least 2 organs, e.g. bone + liver)	
All 3 adverse factors present	9
Two of these factors present	42
One of these factors present	36
No adverse factors present	13
LabBM score (5 blood test results)	
LabBM score 0 (favorable)	15
LabBM score 0.5	7
LabBM score 1.0	17
LabBM score 1.5	21
LabBM score 2.0	14
LabBM score 2.5	15
LabBM score 3.0	9
LabBM score 3.5	2

*LDH* lactate dehydrogenase

^*^progressive after previous treatment or not yet treated

^**^examples uncontrolled primary tumor or liver metastases, irrespective of number and size

The cause of death was unrelated to brain metastases in 61%. Both, extracranial metastases and uncontrolled primary tumors leading for example to hemoptysis or refractory pneumonia were among the documented causes of death. Brain metastases may have contributed to death in 32% (uncertainty because the patients died at home or in nursing homes; no firm documentation about the last days in our electronic patient records; both intra- and extracranial tumor activity was recorded before hospital care was terminated). Definitive confirmation of brain-related death was available in the remaining 7%, including one patient who died from hemorrhage. Treatment-related death (grade 5 toxicity) did not occur. Completely unexpected death was not observed, e.g. accident or suicide.

Univariate analyses (all factors included in Table 1 were tested; chi-square tests) revealed numerous risk factors for 30-day mortality, which were carried forward to confirmatory regression analysis. The predictive factors that achieved statistical significance in the logistic regression analysis are shown in Table 2. Based on these 9 factors (each assigned 3–6 points), a point sum was calculated for each patient. The point sum ranged from 0 (no risk factors for death within 30 days present) to 30. The results can be grouped into 3 or 4 risk categories, as displayed in Table 3. Because the model did not perform optimally (Harrell’s C 0.68; only 10 cases of 30-day mortality were assigned to the highest risk group; 10 of 28), we provided a complete data overview by tabulating the baseline parameters of all 28 patients who experienced 30-day mortality in Table 4. As illustrated in the table, 4 of 28 patients (14%) had less than two risk factors. Among them was a 93-year-old patient with uncontrolled lung cancer and hepatic metastases, whose early death would not be considered surprising by most oncologists. This example illustrates that combining a statistical model with oncological experience may be a reasonable approach.

**Table 2:** Factors predicting 30-day mortality (*p* < 0.05 in multinominal logistic regression analysis)

Parameter	Percent 30-day mortality	Points
LabBM point sum ≥ 3	55	6
Karnofsky performance status (KPS) 50	57	6
Cancer type*	64	6
Extracranial metastases > 3 organ systems**	45	5
Extracranial metastases 3 organ systems***	40	4
Bone metastases present	41	4
Uncontrolled primary tumor	38	4
KPS 60	33	3
Number of brain metastases > 3	31	3

^*^bladder, gastrointestinal none-colorectal, breast hormone receptor positive Her2 negative

^**^example liver, lung, bone, adrenal glands

^***^example skin, peritoneum, pleura

**Table 3:** Point sum leading to the final prediction model

Point sum	Number of cases	Percent 30-day mortality
0–8	3/43	7
9	1/3	
10	2/6	
11	3/10	
12	0/2	29 (9–12 points combined)
13	3/7	
14	5/10	
15	0/3	
16	1/4	38 (13–16 points combined)
17	2/3	
18–30	8/9	83 (17–30 points combined)

The two patients with 17–30 points who survived beyond 30 days died after 1.9 and 2.0 months, respectively

Harrell’s C of 0.68 was higher than that of LabBM alone (0.61) and EC-S alone (0.60)

**Table 4:** Factors indicating poor prognosis (bold text) in all 28 patients who died within 30 days. Typically, at least twofactors were present, e.g. poor performance status and numerous brain metastases. Four patients had less thantwo factors

Cancer type	RT	Incomplete	PT control	Non-brain metastases	Active sites incl. primary	KPS	Number (brain)	Symptoms	Int (mo.)	Age (yrs.)	EC-S	LabBM	OS (mo.)	Cause of death	Factors <2
Esophagus	WB30	1	1	hep, oss, lym	**3**	**6**	3	1	6	70	2	2.5	0.3	extracran	
Melanoma	WB30	0	**0**	pul, hep, lym	**4**	**6**	**5**	1	0	62	1	0	0.8	intracran	
NSCLC	WB30	1	**0**	pul, hep, adr, oss, lym	**5**	8	2	1	0	69	1	0	0.4	extracran	
Jejunum	WB20	0	**0**	pul, hep, adr, oth	**5**	7	**6**	1	0	67	3	**3**	1.0	extracran	
NSCLC	WB20	0	**0**	pul, adr, oss, lym	**5**	**5**	2	0	0	63	3	**3**	0.5	extracran	
NSCLC	WB20	0	**0**	0	1	**6**	**4**	1	5	69	1	1.5	0.4	unk	
NSCLC	WB20	0	1	oss, adr, oth	**3**	**5**	**7**	1	3	64	2	2.5	0.6	extracran	
Kidney	WB30	1	**0**	hep, oss, adr, pul	**5**	**6**	**5**	1	2	56	2	2	0.6	unk	
NSCLC	WB30	0	1	pul	1	**5**	**6**	1	3	55	1	1.5	0.8	unk	
Kidney	WB30	1	**0**	oss, pul	**3**	**6**	**4**	1	35	63	2	1.5	0.1	extracran	
NSCLC	WB30	0	**0**	pul	**2**	**6**	**4**	1	4	66	2	**3**	0.7	extracran	
ER + Her2 -	WB30	0	1	pul, lym, adr	**3**	7	**7**	1	70	67	1	0	1.0	extracran	
NSCLC	WB30	0	**0**	adr, oss	**3**	7	**17**	1	0	53	2	1	1.0	extracran	
NSCLC	WB20	0	**0**	hep, adr, lym	**4**	**5**	**4**	0	5	65	3	**3**	0.7	extracran	
Bladder	WB30	0	1	oss, adr, lym	**3**	7	2	1	38	74	1	1.5	0.7	unk	<2
NSCLC	WB20	0	1	pul	1	7	**4**	1	22	65	1	1.5	0.5	extracran	<2
Melanoma	WB30	0	1	pul, oss, lym	**3**	7	**18**	1	8	**77**	2	1	1.0	intracran	
Bladder	WB30	0	**0**	pul, oss, lym	**4**	**6**	**9**	1	25	75	1	2	1.0	extracran	
Melanoma	WB30	0	1	pul, lym, ski	**3**	**5**	**6**	1	6	55	3	1.5	0.7	unk	
NSCLC	SRS	0	1	Pul	1	7	1	1	3	75	1	2	0.8	extracran	<2
ER+ Her2-	WB30	1	1	hep, pul, oss, oth	**4**	**5**	**12**	1	154	74	3	**3**	0.3	unk	
SCLC	CTx	1	**0**	Oss	**2**	**6**	**50**	1	0	82	0	0	0.1	unk	
Esophagus	WB30	1	1	lym, adr, oss	**3**	**5**	**8**	1	6	72	2	1	0.1	unk	
NSCLC	SFRT	0	**0**	hep, oss, pul	**4**	**5**	1	1	0	66	3	**3**	0.7	extracran	
NSCLC	SFRT	1	**0**	hep, pul	**3**	7	1	1	0	93	2	2.5	0.1	extracran	<2
Rectum	WB30	1	**0**	pul, lym, oth	**4**	**6**	**10**	1	10	48	1	1.5	0.1	extracran	
NSCLC	WB30	0	**0**	0	1	**6**	**5**	1	0	76	0	0.5	0.7	extracran	
NSCLC	WB30	0	**0**	hep, oss	**3**	7	**4**	1	18	51	2	2	0.7	extracran	

Bold text is utilized to identify those parameters that indicate a poor prognosis. Regular text indicates parameters unrelated to prognosis

*RT* radiotherapy, *PT* primary tumor, *KPS* Karnofsky performance status, *Int* time interval between cancer diagnosis and brain metastases, *EC-S* extracranial score, *LabBM* LabBM score, *OS* overall survival.

*NSCLC* non-small cell lung cancer, *ER + Her2-* breast cancer (estrogen receptor positive, Her2 negative), *SCLC* small cell lung cancer, *WB30* whole-brain radiotherapy 30 Gy, *WB20* whole-brain radiotherapy 20 Gy, *SRS* radiosurgery, *CTx* chemotherapy, *SFRT* stereotactic fractionated radiotherapy, *hep* liver, *oss* bone, *lym* lymphatic, *pul* lung, *adr* adrenal glands, *oth* other, *ski* skin, *unk* extracranial progression, but brain metastases might have contributed as well.

## Discussion

After more than a decade of partially successful attempts by our group to develop and validate models that predict short survival after treatment of brain metastases, the present study represents a rigorous effort with modified methodology. We increased the number of evaluated variables, selected a narrowly defined cohort of patients with maximum survival of 3 months, and focused primarily on a dichotomized outcome (30-day mortality yes/no), which undoubtedly represents very short survival. We hoped that an in-depth analysis of a limited number of real-world patients treated with different standard approaches might pave the way towards clinically applicable risk stratification, provided external validation of the resulting model will be successful.

As demonstrated in the Results section, 30-day mortality is a highly multifactorial event. Patient-, intra- and extracranial disease-related risk factors were identified, e.g. KPS, number of brain metastases, pattern and extent of extracranial metastases, and blood test results. Interestingly, age was not associated with 30-day mortality, despite its well-known prognostic impact in analyses that looked at complete Kaplan–Meier curves [3, 4]. Given that the model did not identify or explain all instances of 30-day mortality, the real picture is probably even more complicated. This is also illustrated by the example of the 93-year-old patient included in Table 4. Reality might in fact be too complex to replace clinical judgement by partially helpful models. On the other hand, a large proportion of patients in the highest risk group (> 16 points) died within 30 days, and none survived for more than 2 months. Therefore, the model could be regarded as one of several components of decision making. As also evident from Table 4, no more than two of these 28 early deaths can be considered relatively unexpected. Causes such as accident, suicide or sudden cardiac death were not recorded.

It is also important to realize that 30-day mortality rarely was caused by the brain metastases themselves, although a certain number of patients had causes of death that remained difficult to assign. Only 5 of 100 patients did not harbor active extracranial disease, while more than 50% had at least 3 sites. In this context, one should note that we did not account for the number and size of organ lesions. Both, single bone metastases and widespread involvement were grouped under the same label (bone metastases present). Maybe, a more nuanced assessment would improve the predictive model. On the other hand, there is reason to believe that the LabBM score reflects the extracranial disease burden [16]. As suggested from our regression analysis, several measures of extracranial disease activity contributed relevant information.

A different group conducted a retrospective study of patients evaluated for palliative radiotherapy (different indications) from 2017 to 2019 who died within 90 days of consultation [20]. Data were collected for the TEACHH and Chow models and one point was assigned for each adverse factor. The TEACHH model included primary site of disease, PS, age, prior palliative chemotherapy courses, hospitalization within the last 3 months, and presence of hepatic metastases. The Chow model included non-breast primary, site of metastases other than bone only, and PS. A total of 505 patients with a median overall survival of 2.1 months were studied. Based on the TEACHH model, 2%, 77% and 21% were predicted to live > 1 year, > 3 months to ≤ 1 year, and ≤ 3 months, respectively. Utilizing the Chow model, 21%, 50% and 29% were expected to live 15.0, 6.5, and 2.3 months, respectively. Thus, neither model correctly predict prognosis in a patient population with a survival < 3 months.

External validation of our results in a larger study is necessary, given that some of the findings are surprising and based on small numbers. For example, breast cancer patients with hormone receptor-positive Her2-negative disease were at high risk, while those with triple negative disease were not. Accidental findings and overfitting of data are of concern as long as validation results are lacking. In addition, limitations include the single-institution design and the uncertainty about the cause of death in a proportion of patients. For validation studies, it would also be desirable to include intracranial tumor volume and additional surrogate markers of poor survival, e.g. hypercalcemia or cancer-related pericardial effusion or ascites. There are different ways of measuring radio- or chemotherapy utilization near the end of life, e.g. 30-day mortality calculated from start of treatment, 30-day mortality calculated from end of treatment, or treatment in the last 30 days of life. Regardless of this study’s limitations and the unique patient selection, the topic of active treatment in the terminal phase of cancer continues to be important for patients and providers alike [21–24].

## Conclusion

Extracranial tumor progression was the prevailing cause of 30-day mortality and few, if any deaths could be considered relatively unexpected when looking at the complete oncological picture. We were able to develop a multifactorial prediction model. However, the model’s performance was not fully satisfactory and it is not routinely applicable at this point in time, because external validation is needed to confirm our hypothesis-generating findings.

## Data Availability

The dataset supporting the conclusions of this article is available at request from the corresponding author, if intended to be used for meta-analyses.

## Abbreviations

PS: Performance status
DS-GPA: Diagnosis-specific graded prognostic assessment
EC-GPA: Extracranial-graded prognostic assessment score
WBRT: Whole-brain radiotherapy
SRS: Stereotactic radiosurgery
LDH: Lactate dehydrogenase
NSCLC: Non-small cell lung cancer
